# Weeks of life lost to COVID‐19, the case of the United States

**DOI:** 10.1002/iid3.661

**Published:** 2022-06-20

**Authors:** Cuong Vu Manh

**Affiliations:** ^1^ Institute of Research and Development Duy Tan University Da Nang Viet Nam

**Keywords:** COVID‐19, deficit deaths, moving excess‐deficit mortality model, weeks of life lost

## Abstract

**Background:**

Years of life lost (YLL) is a preferable indicator to assess the mortality impact of COVID‐19. This indicator still has limits, however. Therefore, a new approach and its early‐death weeks (eDW) index has been recently proposed to alter YLL. This study aims to add a new approach, the moving excess‐deficit mortality model, and its method, the weeks of life lost (WLL) index. The new method was then used to measure WLL associated with COVID‐19 in the United States (US).

**Methods:**

The natural mortality law and the random pattern of spreading COVID‐19 were employed to support calculating WLL. The natural mortality law implied that under the same living conditions and the weaker would die earlier. The random spreading of COVID‐19 assumed that COVID‐19 causes the weekly number of early deaths in equal proportions from all of those who would have died eventually distributed through the pandemic.

**Results:**

From Week 02 of 2020 to Week 44 of 2021, we found that the US population has lost 56,270,300 weeks to COVID‐19; the average WLL per COVID‐19‐related death is 74 or 1.4 in the unit of years.

**Conclusions:**

The results do not depend on the high heterogeneity of deaths (e.g., age, gender, health status) and on whether COVID‐19 is the main cause of death. The moving excess‐deficit mortality model and WLL index can be applied promptly at any time and anywhere once excess deaths occurred during the pandemic. The index also provides critical insights into COVID‐19, which can support making public health policies and decisions.

## Graphical abstract

A new approach, the moving excess‐deficit mortality model, and its method, the weeks of life lost to measure the mortality burden of COVID‐19 in the United States.

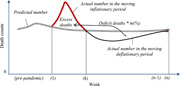

## 1 INTRODUCTION

A number of studies have employed the *years of life lost* (YLL)^1^ method to calculate the mortality burden of COVID‐19.^2^, ^3^, ^4^, ^5^, ^6^, ^7^, ^8^ These studies take into account the heterogeneity in terms of ages, gender, and health conditions for each COVID‐19‐related death, which differ from the excess mortality method. Remarkably, Ferenci^9^ has used the available data on an individual level on comorbidities in Hungary to calculate the YLL associated with COVID‐19, which reports a result differing significantly from that of other studies, is 1.3 years compared to 16 years estimated by Pifarré et al.^2^ for per death after adjusting for 11 comorbidities.

However, in a recent study, Cuong^10^ argued that the YLL and adjusted YLL methods above still have methodological limits, and proposed an alternative approach and index, the early‐death weeks (eDW). Accordingly, calculating the eDW index can provide a more precise measurement of the COVID‐19 mortality because it can avoid five effects: environmental changes and medical/healthcare progress; the heterogenous life expectancy across the populations; the inconsistent and heterogenous historical statistical data of the mortality of severe diseases across countries/geographies; the limited number of comorbidities is taken into account; the COVID‐19 death of people whose death certificate indicates COVID‐19 as the only cause mentioned.

This study aims to add a new method to the calculating of the eDW index now called the *weeks of life lost* (WLL), meaning the average number of weeks a person has lost of their week‐life expectancy. To do so, we first develop a new model, named the *moving excess‐deficit mortality* model and distinguishes its analysis of mortality of the COVID‐19 pandemic from others by focusing on its ongoing manifestation and two crucial terms: *early deaths* (was first used by Cuong)^10^ and *deficit deaths*, especially elaborating the relationship between these terms and excess deaths. While the early deaths mean people who died earlier due to COVID‐19,^10^ the deficit deaths reflect a shortfall of the predicted number of deaths in a given period of the pandemic. Next, we further develop a pattern of the proposed model, namely the *excess‐deficit mortality pattern* to support measuring and assessing the mortality associated with COVID‐19, especially valuing the average number of the WLL of early deaths. Finally, this new method is used to estimate the COVID‐19 mortality burden in the United States.

## 2 METHODOLOGY

### 2.1 Modeling

We first exploit the statistical analyses on the COVID‐19 associated deaths which are majorly from the most vulnerable people (see Supporting Information for more details) to recall the *natural‐mortality law* that was also first mentioned by Cuong meaning that under the same living conditions, the more vulnerable people would die earlier once infected with COVID‐19.^10^ More specifically, due to COVID‐19, the most vulnerable people who would have died in the future will die earlier or precisely a numerous weekly predetermined deaths have been brought forward.

Logically, the COVID‐19 pandemic would endure from the *moving inflationary period* to a latter one called the *moving deflationary period* regardless of whether COVID‐19 causes a single wave or multiple waves. We also argue that due to COVID‐19, the actual weekly number of deaths is fewer than the predicted number in the moving deflationary period, so, this deficiency can be referred to as deficit deaths implying those deaths died earlier than expected (see Supporting Information for more details).

**Figure 1:**
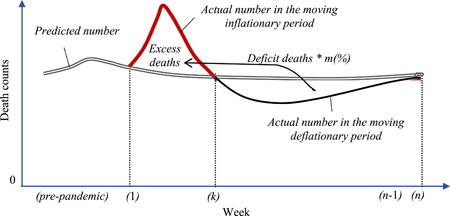
The moving excess‐deficit mortality model of COVID‐19. *m* is an adjusted coefficient in Exp. (2) as $\sum_{w\;=\;1}^{k}E_{w}^{\mathrm{virus}}\;=\;m\;\times \;\sum_{w\;=\;k\;+\;1}^{n}D_{w}^{\mathrm{virus}}$ and estimated based on the data of the most vulnerable people who live longer than usual or shorter than expected (see Supporting Information for more details). The value of *m* approaches 100% (or the value of one) as more deaths of the most vulnerable people are included in the calculation according to the law of large numbers.^6^

In a global context, the current pandemic is not actually ending its moving inflationary period at a specific week due to the spread of the new variants of COVID‐19. Thus, a specific week that is denoted by *k* may be defined as the ending week of a certain wave while it is still in the moving inflationary period of other waves, and week *k, in this case*, can be referred to as a moving observed week. Naturally, the longer the moving inflationary period, the longer the moving deflationary period and the higher value of number *k*, regardless of whether there are several weeks or short periods without excess deaths. Logically, the moving inflationary period will also end at 1 week called *k*th (see Supporting Information for more information) that can be defined for several situations, such as the ending week of the pandemic or of a specific wave in a certain country or of the world. Figure 1 models all of the above‐mentioned properties and logic graphically.

The graphic above shows several main points that require further explanations. First for this specific case, the number of excess deaths in Week *k*, $E_{k}^{\mathrm{virus}}$, gradually declines to zero, such as at the end of this week for simplicity and indexed by *K* before shifting into the moving deflationary period. In reality, this *K*‐point varies from country to country and drops at any week during the pandemic; it depends not only on the levels of nonpharmaceutical interventions (NPI) but also on the timing of the imposition (e.g., soon or late). For example, countries with a higher ratio usually enter the deflationary period earlier, such as Italy with 99.2%.^11^ In contrast, countries with a lower ratio can reach the deflationary period later, such as Sweden with 93% and the United States with 92.4%.^12^, ^13^ Otherwise, countries that quickly imposed strict lockdowns, such as the United States and the United Kingdom, could face multiple waves earlier before moving into the deflationary period.

Second, while both the numbers of excess deaths and deficit deaths are equal, their respective time periods during which the deaths occur may not be the same. In particular, the number of weeks from Week *k* + 1 to *n* is fewer than that from Week 1 to *k* in many situations, such as when NPIs are strictly followed, herd immunity is widespread, and the vaccine is available. Conversely, the moving deflationary period will be longer than the moving inflationary period. And third, although the model can be applied to any country in the world, its shapes and features are not the same. For instance, the older population of Italy will be different from the younger population of the United States. Further, the model can also be applied in multiple simultaneous outbreaks, such as seasonal flu.

Understanding the COVID‐19 early deaths requires the incorporation of the natural mortality law into a given random spread of COVID‐19. Logically, this incorporation leads to an assumption that COVID‐19 causes the weekly number of early deaths in equal proportions among all those who may be weekly predetermined deaths through the pandemic periods. From which we use the moving inflationary‐deflationary periods of the pandemic to constitute a distribution of early deaths, the excess‐deficit mortality pattern. The additional details on this pattern are available in Supporting Information. Subsequently, we used the methodology above combined with the data related to COVID‐19 in the United States to calculate the average WLL of early deaths.

### 2.2 Data strategies

To calculate WLL we first have to estimate the total number of early deaths from the data on excess deaths associated with COVID‐19. These data were retrieved from the Center for Disease Control and Prevention (CDC) of the United States from Week 13 of 2020 to Week 44 of 2021.^14^ Next, we summarize two other indicators: death counts and excess deaths, especially YLL to enable comparison. The data on COVID‐19 deaths (or deaths with certification and coding of COVID‐19 as the cause of death) were retrieved from the CDC in the same period as Week 44 of 2021,^15^ which provides detailed information on the weekly number of deaths and excess deaths.

Although data on both excess deaths and COVID‐19 deaths were retrieved from the same source and as of Week 4, 2022, there are some minor differences between the two. First, while the first death involving COVID‐19 was confirmed in Week 2 of 2020, the excess deaths were only estimated since Week 13 in the same year. Second, the total number of deaths by all causes, from which the estimated weekly number of excess deaths (Supplementary Data_S1) is higher than that of the other source (Supplementary Data_S2), with 5,615,826 compared to 5,608,279 deaths, respectively. The difference of 7547 deaths accounts for 0.1% of the total deaths. Third, the data were collected through Week 4 of 2022, but the calculation was only used till Week 44 of 2021 (ending on June 6, 2021) because the data for the last 11 weeks are incomplete at the time this study was conducted.^15^ Thus, the *K*‐point was defined at the end of Week 44 of 2021 and takes the value of 85 for the entire ongoing pandemic, which means that Week 85th is also the observed week (see Supporting Information for more information).

## 3 RESULTS

From Week 2 of 2020 to Week 44 of 2021 in the United States, COVID‐19 has caused 56,270,300 WLL of 882,886 early deaths. The former reflects the overall mortality burden of the COVID‐19 pandemic in units of weeks or the cumulative WLL of the population while the latter is a proper result than the number of death counts and excess deaths. And each of the COVID‐19 early death has lost an average of 74 WLL. These results together with other conventional metrics were tabulated in Table 1 for convenient comparison.

**Table 1:** The mortality burden of the COVID‐19 pandemic as of Week 44 of 2021, the United States

The mortality of COVID‐19	Early deaths^a^	Early‐death weeks^b^	Weeks of life lost^c^	COVID‐19 deaths^d^	Excess deaths^e^	Years of life lost^f^
Total/average estimated number	882,886	56,270,300	74	775,120	875,772	16

Abbreviations: WLL, weeks of life lost; YLL, years of life lost.

^a^ The cumulative number of early deaths was calculated by Exp. (5).

^b^ The cumulative number of early‐death weeks was calculated by Exp. (7) with the results estimated from the previous step by Exp. (6).

^c^ The average number of WLL was calculated by Exp. (9) with the results estimated from the previous step by Exp. (8) (see Methods and Supporting Information for the detailed specifications). The first three metrics are calculated directly from the number of excess deaths, which is provided based on comparing the observed numbers of deaths with historical trends from 2013 to the present. This average expected number of deaths is straightforward; thus, no confidence intervals are available (see Supplementary Data_S1).

^d^ The total number of COVID‐19 deaths was aggregated from Supplementary Data_S2.

^e^ The total number of excess deaths was aggregated from Supplementary Data_S1.

^f^ The average result calculated by the YLL method.^2^

Table 1 provides a comprehensive overview of and clarifies the distinction between two key indicators related to the ongoing pandemic: COVID‐19 deaths, excess deaths, and early deaths. Accordingly, the cumulative total number of COVID‐19 deaths through Week 85 (or 44 of 2021) is the lowest, just about 88.5% of the cumulative number of excess deaths denoted by $E_{\mathrm{sum}\;\to \;85}^{\mathrm{virus}}$ and 87.8% of the cumulative number of early deaths denoted by ${\mathrm{eD}}_{\mathrm{sum}\;\to \;85}^{\mathrm{virus}}$. This probably has two situations where there were no adequate records of all deaths associated with COVID‐19 or the weekly number of excess deaths was overestimated. Otherwise, the difference between ${\mathrm{eD}}_{\mathrm{sum}\;\to \;85}^{\mathrm{virus}}$ and the number of COVID‐19 deaths is quite significant with 107,766 cases. This, in turn, implies a critical problem in tracking the COVID‐19 deaths of those who did not show the symptoms of COVID‐19 disease.

Measuring the cumulative total number of WLL through Week 85 denoted by ${\mathrm{eDW}}_{\mathrm{sum}\;\to \;85}^{\mathrm{virus}}$ is another goal of this study, which reflects the mortality effects of COVID‐19. Accordingly, the table above shows that the value of this index is 56,270,300 weeks, which provides more insights than the values of $E_{\mathrm{sum}\;\to \;85}^{\mathrm{virus}}$ and YLL. First, this value is expressed in units of weeks and reflects the loss of life expectancy in weeks rather than in years of all COVID‐19 deaths. Second, the value does not depend on the heterogeneity of COVID‐19 deaths (e.g., age, gender, and health status) and whether COVID‐19 was the main cause of death to estimate the total WLL of a population. Thus, the index can be used efficiently to assess and compare the damages caused by pandemics among states or countries.

Finally, the key metric for directly comparing to YLL in Table 1 is the average number of WLL denoted by ${\bar{W}}_{\to 85}^{\mathrm{earlydeath}}$ with the value of 74 weeks. This value implies that each COVID‐19 death in the United States has lost 74 weeks of life expectancy or 1.4 years, which is fewer than 11 times compared to the average YLL in 81 countries (including the United States) of 16 years.^2^

## 4 DISCUSSION

This study follows the early‐mortality approach that was proposed by Cuong to further seek an accurate and insightful measurement of life lost to the pandemic as well as the method of calculating WLL, then used it to measure the mortality impact of COVID‐19 in the United States. We found that from Week 2 of 2020 to Week 44 of 2021, COVID‐19 has caused at least 882,886 early deaths with a total of 56,270,300 WLL. The former number is significantly higher than the number of death counts and at least nearly equal to the number of excess deaths. From a statistical analysis aspect servicing health policies, the number of early deaths is crucial in that it exactly reflects how many lives have been lost associated with COVID‐19 regardless of whether COVID‐19 was the main cause of death or not. This indicator also provides a comprehensive assessment of the overall mortality effects of COVID‐19 in units of weeks to avoid the different discounting life expectancies among deaths.

The main goal of the study is to estimate the average number of WLL of early deaths by the new method. This indicator can provide more accurate and rapid metrics for accessing and disseminating information during any week of the COVID‐19 pandemic anywhere. In the United States, we calculated her average COVID‐19‐related WLL is 74 or 1.4 in the unit of years. This value is a particularly low level if compared to the value of 16 years calculated by the YLL method,^2^ and a bit higher than 1.3 years by the adjusted YLL.^9^ Noteworthy, although the method of estimating WLL differs from the one Cuong used for eDW,^10^ both of them are the same in nature to reflect the loss of life expectancy in units of weeks. However, their values in fact vary a bit, 74 WLLs and 70 eDWs, respectively. The reason for this difference is due to their used number of excess deaths that were extracted from two different sources, the CDC and the Human Mortality Database (HMD).^16^

From the pathology perspective, the average WLL critically conveys a maximum shortfall in life expectancy due to COVID‐19 disease; hence, it can compare to other causes of premature mortality (e.g., cardiovascular diseases, traffic accidents). This indicator goes beyond the epidemiological questions and implies a more important social issue in ethnographic and moral aspects. Employing the WLL index provides more comparable and insightful results that could better guide public health decisions. Further, both the total WLL and average WLL can assist the assessment of the *value of a statistical life* ^17^ and economic cost of the US COVID‐19 mortality as well as compare its results to other top diseases or outbreaks.

There is one potential source that may cause up‐or‐down bias to our results, the accuracy of the predicted number of total deaths in the case of no COVID‐19. This number may be an undercount due to not taking into account the different mortality effects of seasonal influenza over years,^18^ which were different between the death counts in 2014 and 2018 compared to other years (see more details in Supplementary Data_S3). As a result, the number of excess deaths may be affected as well.

## CONFLICT OF INTEREST

The author declares no conflict of interest.

## Supporting information

Supporting information.

## Data Availability

All data supporting the findings of this study are available within the article.
