# Supplementary material for: Weeks of life lost to COVID‐19, the case of the United States
Source: Immun Inflamm Dis. 2022 Jun 20;10(7):e661. doi: 10.1002/iid3.661 (PMC9208283; doi:10.1002/iid3.661)
Supplement: Supplementary file 1 — Supporting information. [file IID3-10-0-s001.docx]

Supplementary Information:
Weeks of life lost to COVID-19 in the United States

**This PDF file includes:**

- Methodology: a description of the approach and methods.
- Data sources: on death counts and excess deaths associated with COVID-19, and seasonal influenza deaths.

Methodology

*Bases and assumptions of model*

In the initial months of the COVID-19 pandemic, most published statistic reports and studies such as from Italy, Sweden, and the US shown that COVID-19 often causes death to the elderly (i.e., who are 64 years old and older) and/or to those who having comordities (e.g., hypertensive disease, diabetes).^1-3^ Cuong has called these COVID-19 deaths are from unhealthy people and all other COVID-19 related deaths are from healthy people. [4] Although named differently, these people in nature are the most vulnerable ones, hence, the weekly number of deaths from these most vulnerable people can be also estimated based on mortality rates in the previous years for the same place and time.

According to the *early-mortality* methodology,^4^ due to infection with COVID-19, the most vulnerable people who would have died in the future die earlier, and a portion of these early deaths becomes excess deaths, named $E_{w}^{virus}$ (with *w* = 1, …, *k*) during the moving inflationary period of the pandemic, particularly in the earlier period.

In a global context, the current pandemic is not actually ending its moving inflationary period at a specific week due to the spread of the new variants of COVID-19. Thus, a specific week that denoted by *k* may be defined as the ending week of a certain wave while it is still in the moving inflationary period of other waves, and week *k* in this case can be referred to as a moving observed week. For simplicity, define the point K as the ending point of week *k*th. Due to deaths from COVID-19, a large number of weekly pre-determined deaths have been brought forward into the *moving inflationary period* before the point K, so, there will be a *moving deflationary period*, which is assumed to transpire from week *k*+1 to *n*. During this period, the actual weekly number of deaths is fewer than the predicted number, and this deficiency can be reffered to as *deficit deaths*, indexed by $D_{w}^{virus}$ that implies those deaths died earlier than expected and estimated by the expression

$D_{w}^{virus}={A_{w}^{all}-p}_{w}^{all}$, *w* = *k*+1, …, *n*  (1)

where $A_{w}^{all}$ and $p_{w}^{all}$ are the actual and predicted weekly numbers of deaths by all causes in the moving deflationary period.

As the early deaths have been brought forward vary from a few days to many weeks, regardless of whether COVID-19 is the main cause or not. Thus, considering the entire period from week 1 to *n*, in which, the number of excess deaths in the moving inflationary period from week 1 to *k* is theoretically equal to the number of deficit deaths in the moving deflationary period from week *k*+1 to *n*, means

$\sum_{w=1}^{k} E_{w}^{virus}=\sum_{w=k+1}^{n} D_{w}^{virus}$, *w* = 1, …, *n* (2)

This argument is the most essential for constructing the model. There is always a difference, indicated by *m* (see Figure 1 for more details) between the number of excess deaths and the number of deficit deaths of the most vulnerable people, especially that of the healthy people. That is due to the deaths of the latter do not occur according to the natural mortality law in the same way as those of the former. The season is that in reality, some people who are older or who have serious diseases may live longer than usual or shorter than expected and otherwise, the most vulnerable ones who become infected with COVID-19 on a weekly basis may die a few days to several weeks or even months earlier.^4^ Therefore, according to the law of large numbers,^5^ the value of *m* is not significant.

*Excess-deficit mortality pattern*

Naturally, COVID-19 spreads randomly and causes deaths eventually for all those who would have died during the entire pandemic period. Specifically, there must be a number of early deaths from those who would have died in the nearest weeks and who would have been brought forward at least one week. Conversely, there must also be other early deaths from those who would have died in the last week of the moving deflationary period. For the sake of simplicity, assuming that the last week of this period is defined here as 2*k* (2*k ≤ n*).

Given the random spread of COVID-19, it makes usefullness to assume that COVID-19 causes the weekly number of early deaths indexed by ${eD}_{w}^{virus}$ in equal proportions from all of those who would have died eventually distributed through the pandemic periods. This assumption also supports the estimation of the cumulative number of early deaths during the pandemic and the average number of weeks that all early deaths are brought forward or reffered to as *weeks of life lost* (WLL). The most useful of these estimations is that all estimation require only the data of excess deaths. More specifically, during the ongoing excess death weeks, COVID-19 has caused early deaths at week 1 or ${eD}_{1}^{virus}$ to all those who would eventually have died with an equal number from week 2 to 2*k*, indexed by ${eD}_{1}^{peer}$, which equates to ${eD}_{1}^{virus}/(2K-1)$, whereas K is counting numbers from 1 to N that denotes for week *n*. ${eD}_{1}^{virus}$ corresponds to the number of excess deaths in week 1 or $E_{1}^{virus}$ since there are no deficit deaths in week 1. And the mean number of weeks that ${eD}_{1}^{virus}$ are brought forward is $(2K-1)/2$. Next, early deaths at week 2 or ${eD}_{2}^{virus}$ are those who would eventually have died with an equal number from week 3 to 2*k* or ${eD}_{2}^{peer}$ that equals to ${eD}_{2}^{virus}/(2K-2)$. Where ${eD}_{2}^{virus}$ is a sum of $E_{2}^{virus}$ and ${eD}_{1}^{peer}$ because the latter are deficit deaths in week 2 that were brought forward to week 1. And the mean number of weeks in which ${eD}_{2}^{virus}$ is brought forward is $(2K-2)/2$.

Similarly, early deaths during week 3, ${eD}_{3}^{virus}$ are those who would eventually have died with an equal number from week 4 to 2*k*, ${eD}_{3}^{peer}$ equals to ${eD}_{3}^{virus}/(2K-3)$. In which, ${eD}_{3}^{virus}$ is a sum of $E_{3}^{virus}$ and ${eD}_{2}^{peer}$ since the latter are deficit deaths in week 3 that have been were brought forward to week 2. And the mean number of weeks that ${eD}_{3}^{virus}$ are brought forward is $(2K-3)/2$. This continues in the same way through the excess death weeks to week *k* or the end of the moving inflationary period of the pandemic.

In reality, the number of excess deaths in certain weeks can show negative values, hence, it is necessary to add one principle into the analysis above. For example, if there are fewer than three consecutive weeks with no excess deaths, the excess deaths of the next week are considered to be those of the four consecutive weeks and the calculation continues as usual. Otherwise, the K-point can be also defined as the week right before the deficit weeks and considered as the ending point of a specific wave of the pandemic. Then define the next K-point for the next wave and continue the calculation in the same way as that of the first wave. The following pattern shows a basic illustration of these distributions.

**Table S1.** Excess-deficit mortality pattern

| Actual deaths | The moving inflationary period | | | | | | | The moving deflationary period | | | | | | |  | |
| --- | --- | --- | --- | --- | --- | --- | --- | --- | --- | --- | --- | --- | --- | --- | --- | --- |
| Excess death counts involving COVID-19 |  |  |  |  |  |  |  |  |  |  |  |  |  |  |  |  |
|  |  |  |  |  |  |  |  |  | K |  | *Deficit deaths* | | |  | 2*k* |  |
|  | ***E*_1_** | ***E*_2_** | ***E*_3_** | ***…*** | ***…*** | ***E_k_*_-1_** | ***E_k_*** |  |  |  |  |  |  |  |  |  |
| Predicted death counts under the normal conditions |  | ${eD}_{w}^{peer}$ | | | | | | $=\frac{{eD}_{w}^{covid19}}{2K-w}$ | | | |  |  |  |  |  |
|  |  |  |  |  |  |  |  |  |  |  |  |  |  |  |  |  |
|  |  |  |  |  |  |  |  |  |  |  |  |  |  |  |  |  |
|  |  |  |  |  |  |  |  |  |  |  |  |  |  |  |  |  |
|  |  |  |  |  |  |  |  |  |  |  |  |  |  |  |  |  |
| Week | 1 | 2 | 3 | … | … | *k*-1 | *k* | *k*+1 | *k*+2 | … | … | … | 2*k-*1 | 2*k* | … | *n* |

*Notes*: The area of excess deaths shows the weekly number of excess deaths in the moving inflationary period; The inner area with red-bold line shows an evenly distributed sample of early deaths that contribute to the weekly excess deaths, including the unshaded area that indicates the assumed weekly deficit deaths in the moving deflationary period. K is the end point of observed week *k*.

*Source*: Adjusted and developed from the early-mortaltity pattern of COVID-19.^4^

According to Table S1, the distribution of early deaths in week *w*, which takes values from 1 to *k* in the pattern above and is estimated by the following general expression

${eD}_{w}^{peer}=\frac{{eD}_{w}^{virus}}{2K-w}$ , *w* = 1, …, *k*; 2*k ≤ n* (3)

where ${eD}_{w}^{virus}=E_{w}^{virus}+{eD}_{w-1}^{peer}$.

Exp. (3) is used to calculate in detailed steps for each week from 1 to *k* as follows:

In week 1, we have ${eD}_{1}^{peer}={eD}_{1}^{virus}/(2K-1)$. As mentioned before, there are no deficit deaths at the first week of the pandemic, so, the number of early deaths equals the number of excess deaths

${eD}_{1}^{peer}=\frac{E_{1}^{virus}}{2K-1}$ . (3.1)

In week 2, ${eD}_{2}^{peer}={eD}_{2}^{virus}/(2K-2)$, where ${eD}_{2}^{virus}=E_{2}^{virus}+{eD}_{1}^{peer}$. Hence, ${eD}_{2}^{peer}=(E_{2}^{virus}+{eD}_{1}^{peer})/(2K-2)$. Replace Exp. (3.1) into this expression, we have:

${eD}_{2}^{peer}=\frac{E_{2}^{virus}+\frac{E_{1}^{virus}}{2K-1}}{2K-2}$

or

${eD}_{2}^{peer}=\frac{E_{2}^{virus}}{(2K-2)}+\frac{E_{1}^{virus}}{(2K-2)(2K-1)}$ . (3.2)

Similarly, in week 3, ${eD}_{3}^{peer}={eD}_{3}^{virus}/(2K-3)$, where ${eD}_{3}^{virus}=E_{3}^{virus}+{eD}_{2}^{peer}$. Replace the latter with Exp. (3.2) and then put all results into the former, we have

${eD}_{3}^{peer}=\frac{E_{3}^{virus}+\frac{E_{2}^{virus}}{(2K-2)}+\frac{E_{1}^{virus}}{(2K-2)(2K-1)}}{2K-3}$

or

${eD}_{3}^{peer}=\frac{E_{3}^{virus}}{(2K-3)}+\frac{E_{2}^{virus}}{(2K-3)(2K-2)}+\frac{E_{1}^{virus}}{(2K-3)(2K-2)(2K-1)}$ . (3.3)

This continues in the same way until week *k*, which is described by the full expresstions as follows:

${eD}_{k}^{peer}=\frac{E_{k}^{virus}}{\left( 2K-k \right)}+\frac{E_{k-1}^{virus}}{\left( 2K-k \right)\left[ 2K-\left( k-1 \right) \right]}+\frac{E_{k-2}^{visus}}{\left( 2K-k \right)\left[ 2K-\left( k-1 \right) \right]\left[ 2K-\left( k-2 \right) \right]}+\ldots+$

$\frac{E_{2}^{virus}}{\left( 2K-k \right)\left[ 2K-\left( k-1 \right) \right]\left[ 2K-\left( k-2 \right) \right]\ldots(2K-3)(2K-2)}+\frac{E_{1}^{virus}}{\left( 2K-k \right)[2K-\left( k-1 \right)]\left[ 2K-\left( k-2 \right) \right]\ldots(2K-3)(2K-2)(2K-1)}$ .

Since *k* denotes a positive integer and all denominators represent a sequence of terms, the expression with the factorial function on the right-hand side is then rewritten in the new form:

${eD}_{k}^{peer}=\frac{{\left( 2K-k-1 \right)!*E}_{k}^{virus}}{\left( 2K-k \right)!}+\frac{\left( 2K-k-1 \right)!*E_{k-1}^{virus}}{\left[ 2K-\left( k-1 \right) \right]!}+\frac{\left( 2K-k-1 \right)!*E_{k-2}^{virus}}{\left[ 2K-\left( k-2 \right) \right]!}+\ldots$ $+\frac{\left( 2K-k-1 \right)!*E_{2}^{virus}}{\left( 2K-2 \right)!}+\frac{\left( 2K-k-1 \right)!*E_{1}^{virus}}{\left( 2K-1 \right)!}$

or

${eD}_{k}^{peer}=\left[ 2K-k-1 \right]![\frac{E_{k}^{virus}}{\left( 2K-k \right)!}+\frac{E_{k-1}^{virus}}{\left[ 2K-\left( k-1 \right) \right]!}+\frac{E_{k-2}^{virus}}{\left[ 2K-\left( k-2 \right) \right]!}+\ldots+\frac{E_{2}^{virus}}{\left( 2K-2 \right)!}+\frac{E_{1}^{virus}}{\left( 2K-1 \right)!}$ .

As the set of subsequent terms from 1 to *k* also denotes *w*, this expression is rewriten into a new short form:

${eD}_{k}^{peer}=\left[ 2K-k-1 \right]!\sum_{w=1}^{k} \frac{E_{w}^{virus}}{\left[ 2K-w \right]!}$ . (3.*k*)

This formula is also used to estimate the subtotals for each week from 1 to *k* that make up a sequence, ${\{{eD}_{w}^{peer}\}}_{1}^{k}$. Because of Exp. (3) is ${eD}_{w}^{peer}={eD}_{w}^{virus}/(2K-w)$, the number of early deaths in each week is then calculated by

${eD}_{w}^{virus}=\left( 2K-w \right)\{\left[ 2K-w-1 \right]!\sum_{1}^{w} \frac{E_{w}^{virus}}{\left[ 2K-w \right]!}\}$. *w* = 1, …, *k* (4)

Therefore, the cumulative total of all early deaths through week *w* is calculated by the expression

${eD}_{sum\to w}^{virus}=\sum_{1}^{w} \{\left( 2K-w \right)\{\left[ 2K-w-1 \right]!\sum_{1}^{w} \frac{E_{w}^{virus}}{\left[ 2K-w \right]!}\}\}$. (5)

As mentioned earlier, the mean number of weeks brought forward of early deaths in any given week simply half the number of weeks counted from that week to week 2*k* or is $(2K-w)/2$. Therefore, the total number of early-death weeks of those weekly early deaths is calculated by the expression

${eDW}_{w}^{virus}=\frac{\left( 2K-w \right)}{2}{eD}_{w}^{virus}$

or

${eDW}_{w}^{virus}=\frac{{(2K-w)}^{2}}{2}\{\left[ 2K-w-1 \right]!\sum_{1}^{w} \frac{E_{w}^{virus}}{\left[ 2K-w \right]!}\}$. (6)

The cumulative total number of early-death weeks of all weekly early deaths up to week *w* is then by ${eDW}_{sum\to w}^{virus}=\sum_{1}^{w} {eDW}_{w}^{virus}$ and rewritten in the full form:

${eDW}_{sum\to w}^{virus}=\sum_{1}^{w} \{\frac{\left( 2K-w \right)^{2}}{2}\{\left[ 2K-w-1 \right]!\sum_{1}^{w} \frac{E_{w}^{virus}}{\left[ 2K-w \right]!}\}\}$. *w* = 1, …, *k* (7)

Thus, the average number of weeks brought forward of the weekly early deaths accumulated on week *w*, indexed by $\bar{W}_{\to w}^{earlydeath}$ is then estimated by ${eDW}_{sum\to w}^{virus}/{eD}_{sum\to w}^{virus}$ or in full as follows:

$\bar{W}_{\to w}^{earlydeath}=\frac{\sum_{1}^{w} \{\frac{\left( 2K-w \right)^{2}}{2}\{\left[ 2K-w-1 \right]!\sum_{1}^{w} \frac{E_{w}^{virus}}{\left[ 2K-w \right]!}\}\}}{\sum_{1}^{w} \{\left( 2K-w \right)\{\left[ 2K-w-1 \right]!\sum_{1}^{w} \frac{E_{w}^{virus}}{\left[ 2K-w \right]!}\}\}}$ . *w* = 1, …, *k* (8)

Finally, the average number of weeks brought forward of all early deaths accumulated to week *w* (or WLL - weeks that a person has lost their week-life expectancy), is then estimated as follows:

$\bar{WLL}_{\to w}^{earlydeath}=\frac{\bar{W}_{1}^{earlydeath} + \bar{W}_{\to w}^{earlydeath}}{2}$ . *w* = 1, …, *k* (9)

**Data sources**

The data on the excess deaths associated with COVID-19 was retrieved from the Center for Disease Control and Prevention (CDC) of the US from week 13 of 2020 to week 44 of 2021, a public source: https://www.cdc.gov/nchs/nvss/vsrr/covid19/excess_deaths.htm. The total number of excess deaths was shown in Table 1 in the “Results” section (see details in Data_1).^6^

The data on the death counts associated with COVID-19 was retrieved from the CDC of the US from week 2 of 2020 to week 44 of 2021: https://data.cdc.gov/NCHS/Provisional-COVID-19-Death-Counts-by-Week-Ending-D/r8kw-7aab. The total number of deaths was shown in Table 1 in the “Results” section (see Data_2).^7^

The data on the seasonal influenza deaths was retrieved from the CDC of the US from 2014 2020: https://gis.cdc.gov/grasp/fluview/mortality.html (see Data_3).^8^

References

Onder, G., Rezza, G. & Brusaferro, S. Case-Fatality Rate and Characteristics of Patients Dying in Relation to COVID-19 in Italy. *JAMA* **323**(18),1775-1776 (2020). doi:10.1001/jama.2020.4683

The Public Health Agency [Folkhälsomyndigheten]. Weekly report on Covid-19, week 15 "Veckorapport om covid-19, vecka 15"]” (PDF online). The situation in the world [Läget i världen]. (2020).

Stokes EK, Zambrano LD, Anderson KN, *et al*. Coronavirus Disease 2019 Case Surveillance — United States, January 22–May 30, 2020. *MMWR Morb Mortal Wkly Rep* **69**, 759–765 (2020). doi: http://dx.doi.org/10.15585/mmwr.mm6924e2.

1. Vu M Cuong. Early-death weeks associated with COVID-19: a comparison among France, the UK and the USA, *Journal of Public Health* (2021) fdab396, https://doi.org/10.1093/pubmed/fdab396

Bernoulli, J. Ars Conjectandi: Usum & Applicationem Praecedentis Doctrinae in *Civilibus, Moralibus & Oeconomicis* Chapter 4 (1713) (Translated into English by Oscar Sheynin).

Center for Disease Control and Prevention. Excess Deaths Associated with COVID-19. Provisional Death Counts for Coronavirus Disease (COVID-19). Data sets. https://www.cdc.gov/nchs/nvss/vsrr/covid19/excess_deaths.htm (2020, assessed Febuary 2022).

Center for Disease Control and Prevention. Provisional COVID-19 Death Counts by Week Ending Date and State. Data sets. https://data.cdc.gov/NCHS/Provisional-COVID-19-Death-Counts-by-Week-Ending-D/r8kw-7aab (2020, assessed Febuary 2022).

Center for Disease Control and Prevention. Pneumonia and Influenza Mortality Surveillance from National Center for Health Statistics system. Data sets. https://gis.cdc.gov/grasp/fluview/mortality.html (2020, assessed Febuary 2022).
